# Employability resources of unemployed adults: longitudinal effects of a group career intervention

**DOI:** 10.3389/fpsyg.2025.1470611

**Published:** 2025-01-28

**Authors:** Catarina Luzia de Carvalho, João Marôco, Maria do Céu Taveira, Ana Daniela Silva

**Affiliations:** ^1^School of Psychology, University of Minho, Braga, Portugal; ^2^CUL, Universidade Lusófona, Lisbon, Portugal

**Keywords:** career intervention, employability, unemployed individuals, group modality, face-to-face intervention, online intervention, conditional latent growth curve

## Abstract

**Introduction:**

This study evaluates the efficacy of a group-based career intervention designed to promote employability resources among unemployed individuals conducted in face-to-face and online modalities.

**Methods:**

Employing a longitudinal quasi-experimental design, with two intervention groups and a control group, data was collected pre-, post, and two months post-intervention.

**Results:**

Conditional Latent Growth Curve analysis revealed a significant decline in employability resources over time, mitigated by both intervention groups. Human capital and professional development were key at baseline and follow-up, while social capital and networking were crucial post-intervention. Career identity self-career management, and environmental monitoring resources significantly contributed to employability post-intervention.

**Discussion:**

These findings highlight the intervention’s effectiveness in counteracting the negative trend among the general population, underscoring the critical role of ongoing participation in career development activities for unemployed individuals. More empirical research on this type of initiative is encouraged, along with civil society taking responsibility for addressing the psychological challenges of unemployment.

## 1 Introduction

Unemployment has pervasive implications for economies, social dynamics, and the mental well-being of individuals (Thompson et al., 2017). Beyond financial hardships, unemployment significantly affects psychological well-being and the cultivation of career resources (Blustein et al., 2020; Lent et al., 2022). Economic challenges and varying unemployment rates across nations are influenced by factors such as low educational attainment and regional work patterns (OECD, 2019; United Nations, 2023). This highlights the need for country-specific analyses and targeted policies (OECD, 2020).

Portugal faces unique challenges, with unemployment rates consistently exceeding the OECD average [National Statistics Institute of Portugal (INE), 2024; OECD, 2024]. Studies indicate a mismatch between the qualifications, interests, and expectations of job seekers and the profiles sought by employers (De Menezes and Sciulli, 2022). Common governmental policies, including job creation initiatives, unemployment benefits, and retraining programs, often overlook the psychological impact of unemployment on career development (Blustein et al., 2020). These policies depend on the prevailing context and vary based on the governance approach (Caleiras and Carmo, 2024).

Given Portugal’s socio-economic context, addressing the psychological impact of unemployment is crucial. Innovative methodologies are necessary to empower individuals with employability resources for sustained career development (Blustein et al., 2020). Career interventions, particularly when led by trained psychologists or career counsellors, can be pivotal in assisting individuals in crafting enduring career trajectories (Maree and Fabio, 2018; Blustein et al., 2019).

Career interventions involve a comprehensive set of actions designed to foster career growth and facilitate well-informed career choices throughout one’s lifespan (Chen, 2021). These interventions aid in making informed decisions, implementing those decisions, and deriving benefits from them (Brown and McPartland, 2005). To address these challenges, a psychological career intervention named “Employability and Career Self-Management” was developed in Portugal. Grounded in Social Cognitive Career Theory (SCCT), this intervention aims to enhance employability resources and career self-management skills among unemployed individuals. By focusing on career development, the intervention provides the necessary tools for individuals to reflect on their careers and take proactive steps.

This study examines the variation of employability resources over time for individuals who participated in the intervention, offered in both face-to-face and online modalities. The objectives are to explore changes in employability resources among participants and assess the effectiveness of the intervention in enhancing these resources.

### 1.1 Employability and social cognitive career theory

Employability resources play a pivotal role in addressing the multifaceted challenges of unemployment. Initially defined as active work-specific adaptability (Fugate et al., 2004), the concept of employability has evolved into a consensus of multidimensionality, acknowledging its dependency on both individual attributes and contextual factors (Guilbert et al., 2015; Hora, 2023).

Lo Presti and Pluviano (2015) proposed a comprehensive redefinition of employability as a personal resource encompassing skills, abilities, formal and informal career networks, and the capacity to navigate social environments. This model posits that work experiences, individual dispositions, life circumstances, and external events serve as antecedents, ultimately leading to career success and influencing outcomes such as job search behavior, job satisfaction, and commitment to work (Lo Presti and Pluviano, 2015). Such a comprehensive view positions employability as a dynamic personal resource influenced by diverse life circumstances and career experiences, resonating with broader career theories such as Social Cognitive Career Theory (SCCT) (Lent and Brown, 2013).

SCCT emphasises the interactive roles of individual characteristics such as age, personal resources like career self-efficacy, and external influences such as employment policies (Lent and Brown, 2013). Gunawan et al. (2024) demonstrated the connection between employability and SCCT by illustrating how perceived future employability, self-efficacy, and outcome expectations interact to influence adults’ career behaviors and aspirations. This framework provides a comprehensive understanding of how individuals navigate their career development within a larger system influenced by personal agency and societal factors. Furthermore, unemployment as a phenomenon can also be interpreted through the lens of SCCT, highlighting the interplay between internal and external factors. SCCT has framed various studies that found self-efficacy and other socio-cognitive variables are associated with the ability to cope with anxiety, stress, and depression resulting from unemployment, as well as with the motivation to seek employment among the unemployed, with this association being mediated by socioeconomic factors (Lent et al., 2022). Additionally, SCCT has been useful in designing and testing hypotheses about reemployment factors (Thompson et al., 2017), the personal career management of unemployed individuals (Taveira et al., 2017), and one of the most widely used theories to support interventions with unemployed individuals (Carvalho et al., 2024). This perspective prevents the oversimplification of attributing career outcomes solely to individual attributes, promoting instead an integrated view where responsibility for career development is shared. This broader understanding of employability reinforces the socio-cognitive perspective on unemployment, illustrating how both internal and external factors contribute to these challenges. Individuals can effectively manage their careers by strategically integrating and leveraging various resources in response to these dynamics, emphasising the need for a holistic approach to career development interventions.

Employability as a personal resource underscores the importance of heightened awareness of career goals, expectations, work experiences, and professional networks. This interactive process also involves acquiring skills, identifying opportunities, and overcoming environmental barriers, thereby integrating employability resources into broader career development strategies (Cheng et al., 2020). A positive interaction between the above dynamics should then result in resources that prompt the achievement of a valued career path and improve one’s career development (Di Fabio, 2017; Fuertes et al., 2021; Guilbert et al., 2015; Hora, 2023; Lo Presti and Pluviano, 2015; Lo Presti et al., 2019). This broader perspective positions employability resources as an integral part of career development itself (Cheng et al., 2020). Effective utilisation of these resources supports the creation of proactive career strategies essential for navigating diverse career transitions and challenges, including unemployment (Hora, 2023; Lo Presti et al., 2019).

In this context, employability resources include developing awareness of one’s career identity, assuming personal career management, taking responsibility for professional development, becoming aware of the need to monitor the environment, and engaging in networking activities (Lo Presti and Pluviano, 2015). These developmental processes are crucial during unemployment, contributing significantly to enhanced employability outcomes, such as positive core self-evaluations and a proactive approach (Lo Presti et al., 2019).

### 1.2 Career interventions in the unemployment context

Psychological career interventions represent a vital approach to mitigating the profound psychological impact of unemployment on individuals’ career trajectories (Carvalho et al., 2024). These interventions are designed to equip participants with essential tools, encouragement, and support necessary to navigate the complexities of the job market (Drosos et al., 2021; Panari et al., 2020). Effective interventions for unemployed individuals should be meticulously tailored to cultivate a proactive and resilient mindset (Carvalho et al., 2024), integrating techniques that address behavioral, emotional, and cognitive aspects critical for enhancing employability (Lo Presti et al., 2022).

Guidelines for designing and implementing psychological career interventions are crucial for optimising their effectiveness (Lent and Brown, 2020). These guidelines not only refine intervention content but also inform their implementation and research design. However, despite increasing relevance in evaluating the effectiveness of career interventions (Whiston et al., 2017) and identifying features tailored for the unemployed population (Carvalho et al., 2024), there remains a critical need for empirical evidence supporting the efficacy of interventions with this population (Lent and Brown, 2020).

The evaluation of career intervention efficacy necessitates employing rigorous methods to analyse outcomes, including pre- and post-intervention assessments (Whiston et al., 2017; Lent and Brown, 2020) and long-term evaluations (Perdrix et al., 2012). Follow-up assessments conducted post-intervention provide valuable insights into intervention sustainability, identify ongoing participant needs, and offer opportunities for intervention enhancement (Sadler, 1984). These assessments should incorporate self-reported measures tailored to assess the impact of career resources targeted by the intervention (Lent and Brown, 2020). Moreover, it is imperative to consider the influence of intervention modalities on efficacy research (Carvalho et al., 2024; Santilli et al., 2021). Face-to-face interventions are noted for their direct communication, personalised strategies, immediate feedback, supportive atmosphere, and enhanced participant motivation, establishing robust therapeutic relationships (Herman, 2010). Conversely, online interventions address accessibility barriers (e.g., time, place, cost), promoting inclusivity by engaging diverse audiences (Pordelan and Hosseinian, 2020; Seabra et al., 2018). While evidence supporting online interventions is growing (Nota et al., 2016; Richards and Vigano, 2013; Sampson et al., 2019), further research is needed to fully understand and optimise their effectiveness, particularly in the context of unemployment (Richards and Vigano, 2013; Whiston et al., 2017).

### 1.3 Present study

This study examines the efficacy of an employability career intervention grounded in the Social Cognitive Career Theory (SCCT) approach. The primary objectives are: (1) to analyse the trajectory of changes in employability resources among participants over three assessment points; (2) to assess whether participation in the intervention leads to significant improvements in employability resources; (3) to investigate how participation in different modalities (face-to-face and online) affects the trajectory of employability resources; and (4) to evaluate how each dimension of employability contributes to overall employability at each assessment point.

The study hypotheses are grounded in the demonstrated effectiveness of psychological career interventions among unemployed individuals, as evidenced by previous research (e.g., Maree, 2022; Maree et al., 2019). These interventions have consistently enhanced career adaptability (e.g., Santilli et al., 2021), career self-efficacy (e.g., Drosos et al., 2021), and overall employability (Maree, 2022; Panari et al., 2020; Whelan et al., 2018). However, the necessity to substantiate ongoing funding initiatives and demonstrate to society the significant impacts and advantages of such interventions remains crucial (Blustein et al., 2020). Despite potential initial constraints imposed by participants’ unemployment status on their employability resources (Panari et al., 2020), it is expected that (H1) participation in a career psychological intervention rooted in established guidelines for designing and implementing such interventions (Lent and Brown, 2020), will positively influence the trajectory of these resources over the assessment points, thereby indicating its effectiveness.

Research on career intervention modalities for the unemployed population is emerging, with increasing interest in online interventions (Pordelan and Hosseinian, 2020; Sampson et al., 2019). Contrary to traditional studies suggesting that face-to-face modalities were likely to achieve better outcomes than online modalities (Herman, 2010), recent studies suggest comparable efficacy between face-to-face and online modalities (e.g., Amundson et al., 2018). Therefore, considering the consistency of the intervention adaptation procedures for this online modality (American Psychological Association, 2013; Ordem dos Psicólogos Portugueses, 2020; Pordelan et al., 2018), along with recent positive findings for online modalities (Santilli et al., 2021), it is expected that (H2) participating in the intervention in either the face-to-face or online intervention groups similarly affects the trajectory of employability resources over the three-time points, indicating a similar efficacy between the two intervention modalities.

Given that the intervention aims to support participants in reflecting, exploring, and developing an individual career action plan and recognizing that overall employability is a composite measure influenced by multiple interrelated dimensions (Gamboa et al., 2023), it is crucial to hypothesise the specific contributions of each employability resource at different time points. By integrating the sociocognitive approach (Lent and Brown, 2013) with the model of employability resources (Lo Presti and Pluviano, 2015), the interaction and contributions of employability dimensions in people facing unemployment can be better understood. Studies on the characteristics, personal resources, and external influences of the unemployed (e.g., Lent et al., 2022; Taveira et al., 2017; Thompson et al., 2017) provide empirical support for this integration. This approach highlights that the four employability resources collectively enhance overall employability and can be analysed in light of individuals’ experiences at each time point of the sociocognitive intervention.

For instance, developing and implementing a career plan requires individuals to evaluate their current knowledge, skills, and competencies and identify areas for improvement (Lo Presti et al., 2019). As participants engage in career planning activities, they may become more aware of their professional strengths and weaknesses, leading to a proactive approach to upskilling and professional development. As the development of a career plan is one of the last activities of the interventions, participants may only be ready to engage more deeply with their career plan after the intervention. Therefore, it is expected that (H3) Human Capital and Professional Development (HCPD) resources may contribute significantly to explaining global employability at T2 compared to T0 and T1.

Social relationships play a crucial role for individuals confronting unemployment (Bhat, 2010; Lent et al., 2022). The group format within career interventions facilitates social interactions and fosters the development of professional networks or the importance of having one (Carvalho et al., 2024). Within the intervention, participants leverage these interactions to build social capital, expand their career networks, seek mentorship, and access valuable resources. This process suggests that (H4) Social Capital and Networking (SCN) resources may contribute significantly to explain global employability at T1 compared to T0 and T2.

Participants are expected to clarify their career identities and develop effective self-management strategies, aligning goals with personal values and interests through intervention modules focused on self-awareness (Maree et al., 2019). The first intervention module primarily focuses more on self-awareness, and all other modules were designed to use and reinforce this self-awareness. Therefore, (H5) Career Identity and Self-Management (CISM) resources may contribute significantly to explain global employability at T1 compared to T0 and T2.

Staying informed about labour market trends and job opportunities is crucial for career planning (Lent et al., 2022; Taveira et al., 2017). Developing environmental monitoring skills helps individuals regularly track the professional environment, research job prospects, and adjust their career strategies (Lo Presti et al., 2022). This continuous engagement enhances employability by keeping individuals aware of market dynamics (Littman-Ovadia et al., 2014). Since the second intervention module focuses on reinforcing exploration practices, it is hypothesised that (H6) Environmental Monitoring (EM) resources may contribute significantly to explain global employability at T1 and T2 compared to T0.

The study followed a longitudinal quasi-experimental design, with two intervention groups (face-to-face and online group) and a control group, and the data were collected pre-, post and two months after the intervention.

## 2 Method

### 2.1 Settings and intervention design

The Employability and Career Self-Management intervention was developed by a university in Northern Portugal under the scope of the Careers Project (ALG-06-4234-FSE-000047), a collaborative initiative to promote employability in the Southern region. The university’s ethics committee that developed the study approved the intervention and its evaluation protocol (CEICSH 002/2022).

The intervention goal was to promote reflection and decision-making among unemployed adults regarding their educational, professional, and life paths. This group intervention used a career sociocognitive approach and was designed to support participants in: (1) exploring their employability resources and self-career management skills through the identification, recognition, and reflexion of their strengths, personal resources, and experiences; (2) exploring, accessing, and organising structured and reliable information about their context; and (3) developing an individual career action plan.

The face-to-face intervention consists of a set of six in-person sessions, with each session lasting two hours. These sessions took place biweekly, totaling 12 h of intervention. The online intervention consists of three synchronous sessions, each lasting two hours, within a one-week timeframe, resulting in a total of six hours of intervention. The differences in the intervention structure (i.e., reduction in duration and the number of sessions) were made in order to ensure the quality parameters of online interventions (American Psychological Association, 2013; Ordem dos Psicólogos Portugueses, 2020; Pordelan et al., 2018). Both modalities were conducted by two Psychologists in group settings, with a maximum of 10 participants per group. All professionals received 42 h of training on career intervention with unemployed adults and were trained to deliver this specific intervention. Participants were given the flexibility to choose their preferred session schedule (i.e., morning, afternoon, and evening), and every session happened in the same schedule and location to prompt participant’s commitment. The intervention groups actively participated in the entire intervention, while the control group did not benefit from any kind of career intervention until the end of the research project, where they were then offered the intervention. Intervention’ participants provided their written informed consent for the different phases of the research (i.e., in intervention and evaluation) in accordance with the Declaration of Helsinki, and were informed that the study poses no risk, cost or harm to them.

The intervention sessions followed a fixed structure, organised in three stages: (1) Being ME+: Self-reflection and Personal Values; (2) Engaging with the World: Communication, Exploration, and Personal Accountability; and (3) Taking Action and Moving Forward: Support for Considering Alternatives and Decision Criteria. Table 1 presents a comprehensive overview of the intervention, outlining the specific objectives and activities carried out in each session for both face-to-face and online modalities.

**Table 1:** Intervention overview.

Stages	Online modality	Face-to-face modality	Session goals	Session activities
Being ME+	Session 1	Session 1	Administer the pre-test. Facilitate participants’ understanding of the general aspects of the intervention (e.g., structure, duration, goals). Establish a group contract based on individual reflections to ensure a collaborative and respectful environment. Co-create a code of ethics and promote interaction and collaboration within the group (only in face-to-face modality). Foster participants’ awareness of the importance of self-awareness in the career development process. Enhance participants’ understanding and integration of the concept of career and its relationship with life roles. Support participants in reflecting on allocating of time to different life roles and its impact on overall well-being. Facilitate participants’ contemplation on the level of satisfaction associated with different life roles. Promote personal growth by assisting participants in identifying, recognizing, and clarifying their individual life values. Strengthen personal identity by acknowledging participants’ personal resources, characteristics, and past experiences that shape their sense of self and decision-making abilities.	Pre-test. Participant introductions with expectations and motivations setting. Intervention presentation. Signing and reflecting on the group contract (only in face-to-face modality). “Life Values and My Career”—Part A and Part B.
		Session 2	Raise participants’ awareness about the role of self-awareness in career development Facilitate participants’ identification and exploration of personal attributes and interests that contribute to clarity and of a positive self-concept, including self-appreciation and personal branding, and foster optimism and confidence towards the future. Enhance participants’ recognition of their social support network by guiding them to identify, acknowledge, and clarify their contacts and personal and professional experiences. Enhance participants’ positive attitude towards lifelong learning by valuing individual agency, interactive learning opportunities, and leveraging one’s skills and social support network. Assist participants in setting career goals for each area of their lives, aligning them with their values and aspirations.	“Competence +” “My Career Network” “Goals for Life Roles” “Identity Narrative”
Engaging with the World	Session 2	Session 3	Raise participants’ awareness about the concept of career exploration and other related notions. Empower individuals to explore available resources and opportunities by collecting, analysing, and organising relevant information for future career planning. Enable and raise participants’ awareness of efficient utilisation of new technologies in accessing career-related information. Support participants’ identification and recognition of career information, including occupation, education and training, employment, as economic aspects and the range of available career information resources. Foster participants’ personal agency in information exploration, alternative selection, and opportunity optimization. Support participants’ reflection on information related to the world of education, work, and employment.	“Exploration Planning” Exploring online platforms “My Exploration Summary”
		Session 4	Raise participants’ awareness about career exploration methods and career self-management strategies. Support participants’ deepening knowledge about individual agency impact in adapting to unpredictable and uncertain contexts. Foster participants’ personal agency in information exploration, alternative selection, and opportunity optimization. Foster participants’ assertiveness and effective communication skills regarding beliefs, emotions, career aspirations, and goals in interpersonal, group, and network contexts. Promote participants’ positive attitude towards lifelong learning, recognizing the roles, phases, and life contexts that enhance the learning and development process.	“Alternative Selection” “Identity Narrative” Presentation of curriculum vitae and cover letter templates for reflection at home
Taking Action and Moving Forward	Session 3	Session 5	Raise participants’ awareness about the concept of career planning and the inherent principles of its implementation. Support participants’ delineation of personal goals that enable the optimization of an action plan. Aid participants’ recognition of personal goals and preferred decision criteria that facilitate opportunity optimization (integrating changing employment trends, social needs, and economic conditions into career plans).	“SMARTE Goals”
		Session 6	Raise participants’ awareness about the concept of a career action plan. Empower participants to anticipate barriers and identify available supports in plan implementation. Encourage participants’ consideration of a preferred action plan and alternative plans. Promote participants’ reflection on how the action plan aligns with personal conceptions of work-related dignity and its relationship with other life domains that contribute to personal well-being and life satisfaction. Conclude the intervention sessions and administer the post-test.	“Action Timeline” “Identity Narrative” Post-test.

### 2.2 Participants

**Figure 1:**
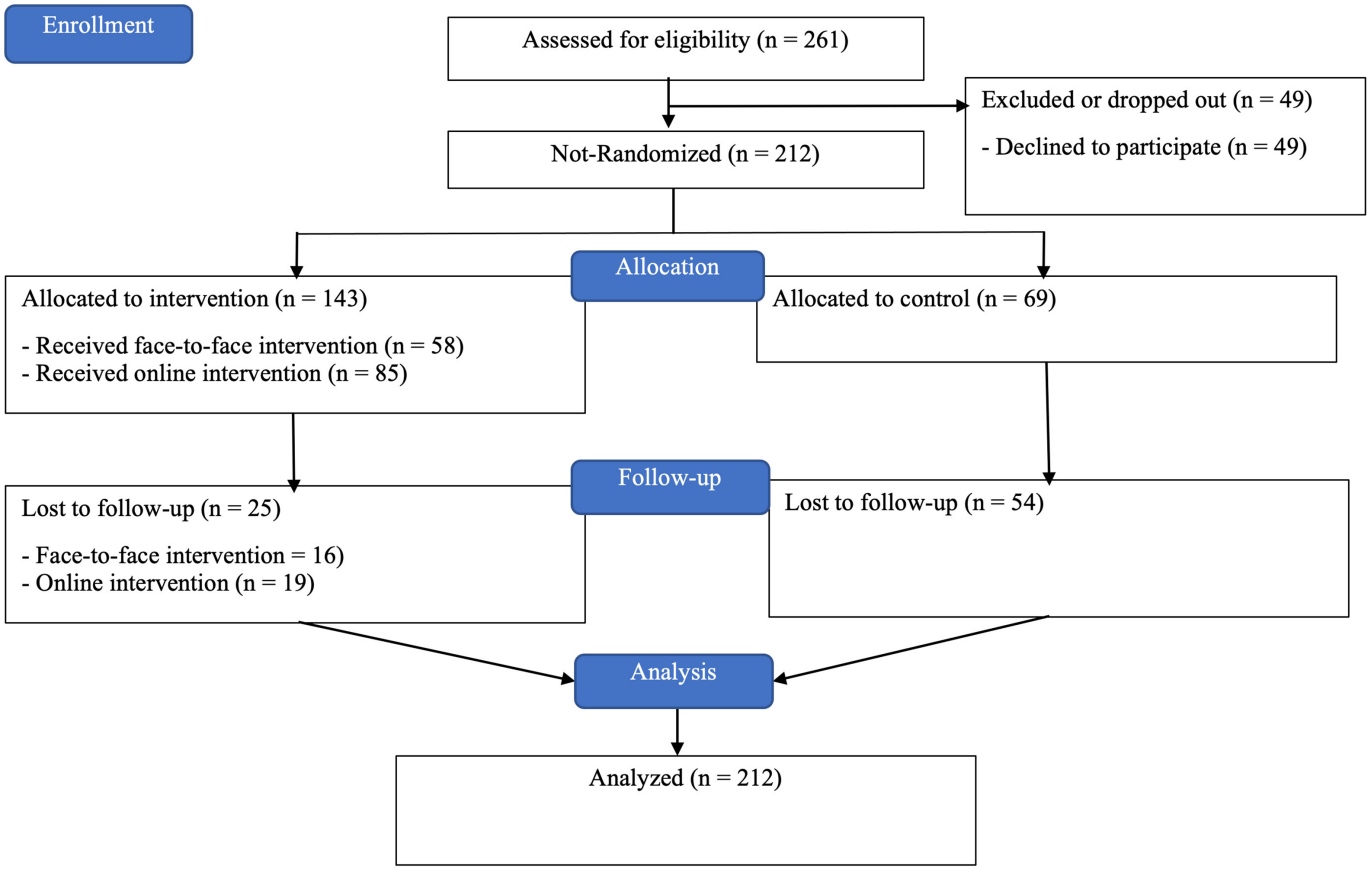
CONSORT flow diagram (Moher et al., 2001).

The career intervention had 212 participants: 58 in the face-to-face intervention group, 85 in the online intervention group, and 69 in the control group. For an overview, see the CONSORT Flow Diagram, Figure 1.

In the face-to-face modality, of the 58 participants, 40 (69%) identified themselves as female and 18 (31%) as male. The participants were aged between 19 and 64 (M = 44.6; SD = 10.319). Of these, 8.5% were aged between 19 and 30, 24% between 31 and 40, 35.9% between 41 and 50 and 30.7% between 51 and 64. With regard to nationality, 52 (89.7%) of the participants were Portuguese, and 6 (10.3%) were Brazilian. In terms of education level, eight (13.7%) had not completed elementary school, nine (15.5%) had completed elementary school, 18 (31%) had completed secondary school, 17 (29.3%) had completed a bachelor’s degree and six (10.3%) a master’s degree.

In the online modality, of the 85 participants, 68 (80%) identified themselves as female and 17 (20%) as male. The participants were aged between 19 and 67 (M = 42.1; SD = 10.291). Of these, 13.2% were aged between 19 and 30, 31.6% between 31 and 40, 34.2% between 41 and 50, and 21.4% between 51 and 67. With regard to nationality, 63 (74.1%) of the participants were Portuguese, 17 (20%) were Brazilian, one (1.2%) was French, one (1.2%) was Colombian, one (1.2%) was São Toméan, one (1.2%) was Venezuelan and one (1.2%) was Guinean. In terms of education level, three (3.5%) had completed elementary school, 36 (42.4%) had completed secondary school, 42 (49.4%) had completed a bachelor’s degree, three (3.5%) a master’s degree and one (1.2%) a doctorate.

The control group consisted of 69 participants. Of these, 54 (78.3%) identified themselves as female and 15 (21.7%) as male. The participants were aged between 20 and 67 (M = 39.5; SD = 10.513). 21.3% were aged between 20 and 30, 35.9% between 31 and 40, 27.4% between 41 and 50, and 114.2% between 67 and 64. With regard to nationality, 55 (78.3%) participants were Portuguese, 11 (15.9%) were Brazilian, one (1.4%) French, one (1.4%) Italian, one (1.4%) Colombian and one (1.4%) Angolan. In terms of education level, seven (10.1%) had completed elementary school, 25 (36.2%) had completed secondary school, 32 (46.3%) had completed a bachelor’s degree, four (5.8%) a master’s degree and one (1.4) a doctorate.

### 2.3 Data collection

Data collection took place immediately before the intervention (pre-test), immediately after the intervention (post-test), and two months after the intervention (follow-up). The questionnaires were created in an online platform (Qualtrics) and shared with the participants through a link or QR code. The research team confirmed and, if necessary, articulated with the psychologist team to ensure that all participants completed them.

#### 2.3.1 Sociodemographic data

Participants’ gender, age, nationality, and level of education were collected.

#### 2.3.2 Employability resources data

The Multidimensional Measure of Employability (MME) (Lo Presti et al., 2019), validated for unemployed people (Lo Presti et al., 2022; Silva et al., 2023) was used to assess employability resources. The MME original version has 28 items answered on a 5-point scale varying from 0 (‘not at all’) to 4 (‘at all’) (Lo Presti et al., 2019). The MME evaluates employability in terms of (1) Human capital and Professional development, encompassing knowledge, skills, and competencies acquired or potentially acquired through formal and professional training that can be useful in the workplace (Items 1 to 9; e.g., “If needed, I think I could adapt to more complex or demanding tasks than the ones previously done”); (2) Social capital and networking, representing the support individuals can benefit from based on the social relationships they have developed or will develop, given their social skills (Items 10 to 16; e.g., “When a decision within a team must be made, I am usually able to convince other people about the goodness of my proposals.”); (3) Career identity and self-career management, reflecting individuals’ perception of their career (Items 17 to 20; e.g., “I believe I have a clear plan for my career”); (4) Environmental monitoring, referring to individuals’ knowledge and awareness of the job market (Items 21 to 28; e.g., “I’m able to gather useful information about a potential employer before a job interview”). Lo Presti et al. (2019) found good reliability indices among employed people (0.81 < *α* < 0.92), Lo Presti et al. (2022) reported similar results in Italian job seekers (α = 0.94), as well as Silva et al. (2023) with Portuguese unemployed people (0.85 < α < 0.92). In this study, in order to find a good fit to the longitudinal data, the scale was simplified—the goodness of fit and its statistical procedures could be consulted in the data analysis section.

### 2.4 Data analysis

All data analyses were done with the IBM SPSS Statistics, version 29, and with R 4.0 (R Core Team, 2020), through RStudio (R Core Team, 2020) (v. 26).

**Figure 2:**
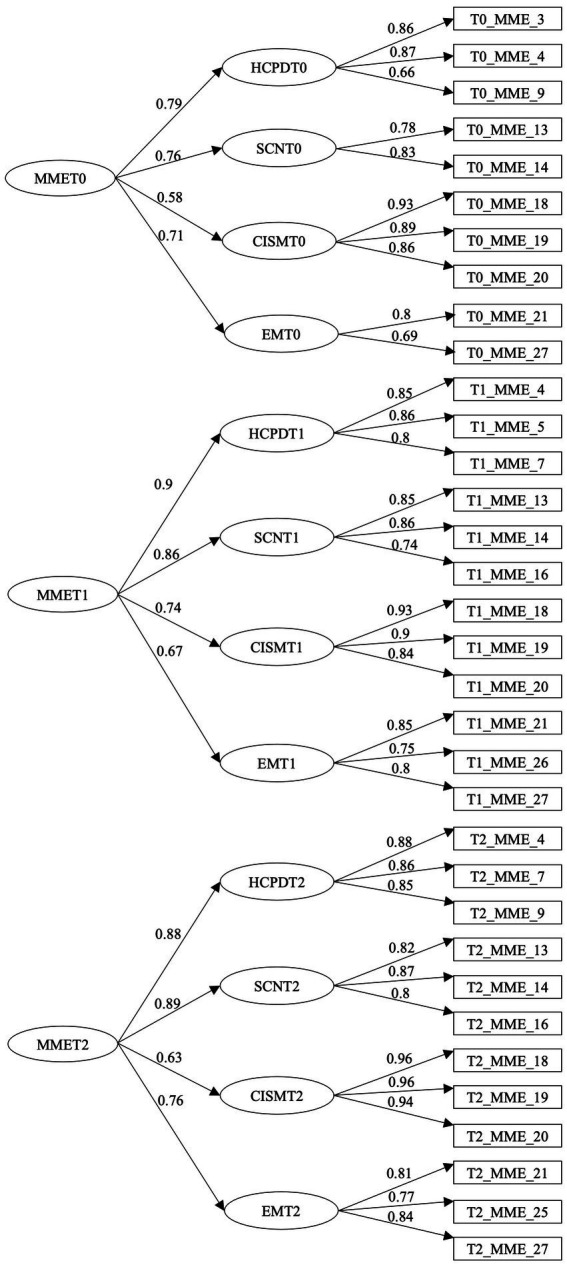
Simplified version of the MME model.

Confirmatory Factor Analysis (CFA) was performed to assess the psychometric properties of the MME (Lo Presti et al., 2019) in the present longitudinal sample. CFA used Robust Maximum Likelihood estimation as implemented in the lavaan package (v.0.6.17) for the R statistical system (Rosseel, 2012). The adequacy of the measurement model was assessed at three different time points using statistical indicators and reference values (CFI and TLI > 0.9; RMSEA ≤0.05; and SRMR <0.08; Marôco, 2021). To achieve a good fit, the first-order factor measurement model was simplified. Thus, respecting the original factor structure, only 3–4 items with the highest factor weight (>0.7) were used per factor (Marôco, 2021). Modification Indices were also used to find significant correlations between items. The simplified model assured the applicability of the MME in the three time points (pre-, post-test, and two months follow-up) for the unemployed sample (𝘟2 (502) = 702.115; CFI = 0.939; TLI = 0.931; RMSEA = 0.057; SRMR = 0.066). The simplified version of the MME model is depicted in Figure 2.

Second, the evolution of employability resources among the participants of face-to-face and online psychological career intervention over three-time points (pre- and post-intervention, and two months follow-up) was evaluated through the specification of a conditional Latent Growth Model (LGM), incorporating longitudinal error correlations with statistical significance at *p* < 0.05 (Marôco, 2021; Spurk et al., 2020). LGMs are a class of models used for modelling longitudinal data within the framework of structural equation modelling to examine inter-individual differences in intra-individual growth trajectories (Marôco, 2021). The LGM incorporated latent intercepts and slopes for each time point (T0, T1, T2) corresponding to the employability indicators, including human capital and professional development (HCPD), social capital and networking (SCN), career identity and self-career management (CISM), and environmental monitoring (EM). The model’s latent intercepts at each time point reflected the initial levels of the employability resources, while the slope indicated the rate of change over time. Specifically, the intercepts and slopes of the MME variables were regressed on themselves and previous time points to capture their evolving trajectories. Moreover, conditional effects were incorporated into the model, wherein the intercepts and slopes of MME were regressed on two predictor variables—the intervention groups (face-to-face and online). These predictors were utilised to explore potential differences in the initial levels and developmental trajectories of employability indicators among distinct participant groups. Additionally, the model accounted for residual covariances between certain latent variables and included the correlation from the intercept to the slope, thereby accounting for additional relationships within the model.

## 3 Results

**Figure 3:**
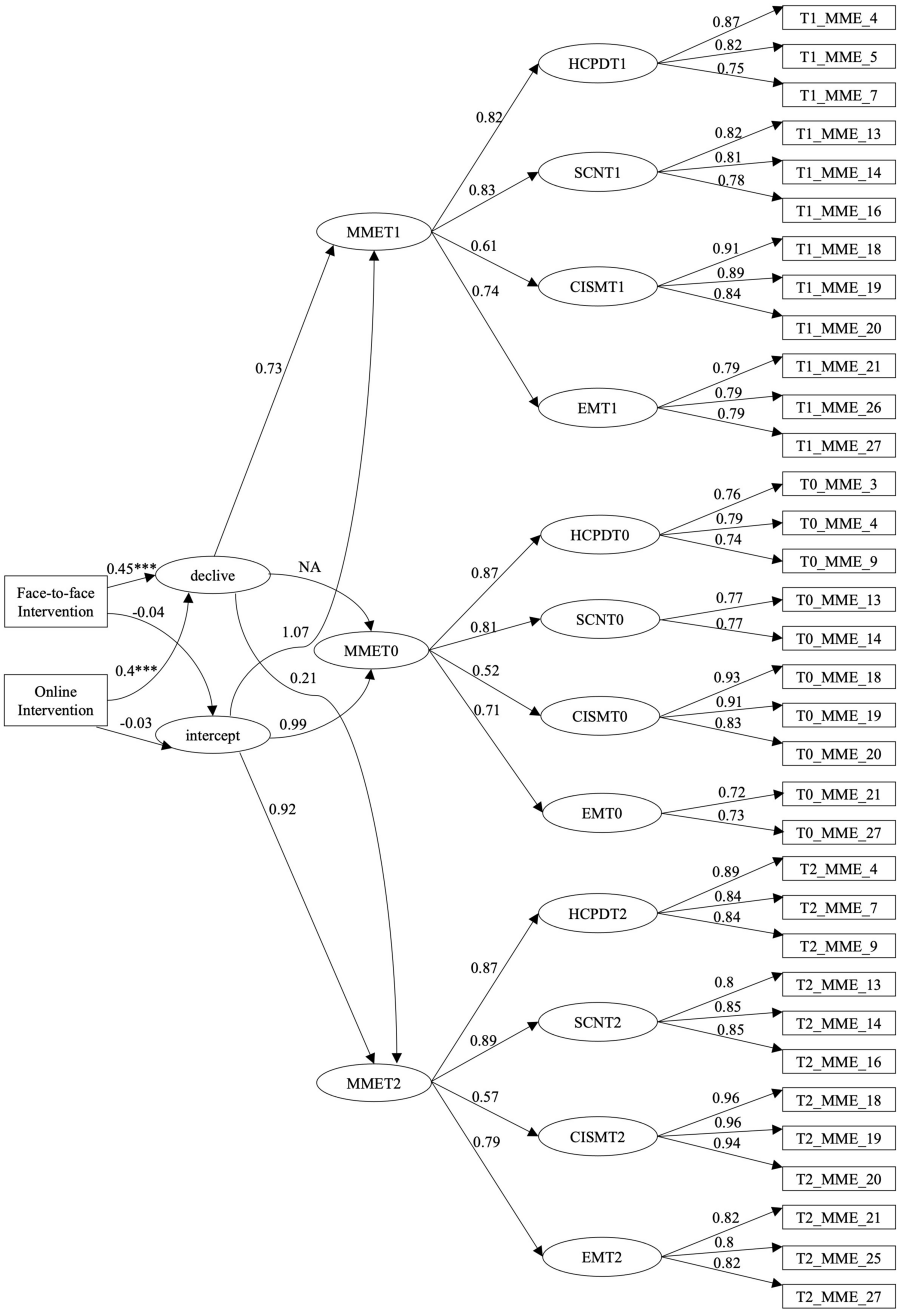
Standardised estimates of the parameters of the conditional model.

In this study, a conditional Latent Growth Curve analysis was employed to examine the longitudinal trajectories of employability indicators, including human capital and professional development, social capital and networking, career identity and self-career management, and environmental monitoring, across three-time points (pre-test, post-test, and two months follow-up) following unemployed individuals’ participation on a career psychological intervention. The LGM revealed an excellent fit to the variance, covariance, and mean structure of the study sample (𝘟2(597) = 929.747; *p* = 0.000; CFI = 0.924; TLI = 0.920; RMSEA = 0.051; SRMR = 0.081). Figure 3 displays the standardised estimates of the parameters of the conditional model.

### 3.1 Trajectory of changes in employability resources

The average intercept was 4.016 (SE = 0.089; *p* < 0.001). This indicates that the baseline employability level significantly differs from the minimum value on the scale (0), showing that all participants start with a certain level of employability resources. Furthermore, the intercept variance was 0.338 (SE = 0.045; *p* < 0.001). This indicates that there was variability among participants in terms of their baseline employability values, indicating that individuals start at different levels of employability resources.

The average slope was −0.240 (SE = 0.088; *p* < 0.001), indicating a negative and significant average decline in employability resources among the study participants. In addition, the slope variance was 0.128 (SE = 0.079; *p* = 0.106). The data shows that the trajectory of employability growth was homogeneous among study participants, indicating that the rate of decline in employability is consistent across individuals. The correlation between the intercept and slope was −0.141 (SE = 0.032; *p* < 0.001), which shows that individuals with a higher baseline value of employability have lower growth of employability.

### 3.2 Efficacy of the two intervention modalities (face-to-face and online)

The conditional LGM results for the intercept variable showed that the predictor face-to-face intervention group variable had an estimated coefficient of −0.057 (SE = 0.119; *p* = 0.631), and that the predictor online intervention group variable had an estimated coefficient of −0.039 (SE = 0.107; *p* = 0.716). This indicates that both face-to-face and online intervention groups have no significant differences on employability resources at the baseline.

For the slope variable, the regression results indicated that the face-to-face intervention variable had an estimated coefficient of 0.397 (SE = 0.105; *p* < 0.001) and that the online intervention variable had an estimated coefficient of 0.323 (SE = 0.097; *p* = 0.001). This indicates that being part of one of the intervention groups, in comparison to being part of the control group, had a significant positive effect on the employability resources trajectory over time factor. These findings highlight the importance of face-to-face intervention and online intervention groups in explaining variations in the changes in employability resources. Therefore, H1, which stated that participating in the intervention would positively influence the trajectory of employability resources over the three time points, is supported by the data. Furthermore, H2, which stated that being part of the face-to-face or online intervention group similarity would explain the changes in the employability resources over time, is also supported.

### 3.3 Employability dimensions strength

The strength of the relationships between the employability resources and their respective indicators (HCPDT, SCNT, CISMT, EMT) in each time point (T0, T1, and T2) reveals that all of them are positive and significant (*p* < 0.001).

For HCPD, the standardised loadings were 0.875 at pre-test, 0.820 at post-test, and 0.872 at 2-months follow-up. Although HCPD resources better explain global employability at T0, when compared with T1 and T2, these resources greatly explain global employability at T2, when compared with T1. Therefore, H3, which stated that HCPD resources would greatly explain the improvements in global employability at T2 compared to T0 and T1, is partially supported by the data.

For SCN, the standardised loadings were 0.810 at pre-test, 0.829 at post-test, and 0.890 at 2-months follow-up. Although SCN resources better explain global employability at T2, when compared with T0 and T1, these resources greatly explain global employability at T1, when compared with T0. Therefore, H4, which stated that SCN resources would largely explain the improvements in global employability at T1 compared to T0 and T2, is partially supported.

For CISM, the standardised loadings were 0.523 at pre-test, 0.611 at post-test, and 0.567 at 2-months follow-up. CISM resources better explain global employability at T1, when compared with T0 and T1. Therefore, H5, which stated that CISM resources would greatly explain the improvements in global employability at T1 compared to T0 and T2, is supported.

For EM, the standardised loadings were 0.710 at pre-test, 0.742 at post-test, and 0.785 at 2-months follow-up. EM resources better explain global employability at T1 and T2, when compared with T0. Therefore, H6, which stated that EM resources would greatly explain the improvements in global employability at T1 and T2 compared to T0, is supported by the data.

## 4 Discussion

The aim of this study was to assess the efficacy of a career intervention designed for unemployed adults, grounded in the Social Cognitive Career Theory (SCCT) framework. This was achieved by analysing alterations in the participants’ employability resources across three distinct time points, with some participants receiving the career intervention either through face-to-face or online modalities. The main objectives of the research were as follows: (1) to track changes in study participants’ employability resources over three-time points (pre-test, post-test, and two months follow-up); (2) to determine if participating in the intervention leads to greater improvements in employability resources; (3) to analyse how participation in each modality (face-to-face and online modalities) affects the trajectory of employability resources for intervention participants; and (4) to examine how each employability resource contributed to overall employability at each assessment point.

The conditional latent growth curve analysis findings indicate a decline in employability resources for all participants, regardless of group assignment. This underscores the pervasive challenges faced by the unemployed population (Drosos et al., 2021) and prompts further investigation into potential explanations and contextual factors driving these trends. Before the intervention, participants exhibited heterogeneous yet generally positive employability conditions. Those with initially robust employability may have had limited opportunities for further enhancement in these resources, particularly without additional stimuli (Hora, 2023). Consequently, some participants may have perceived themselves as already proficient in proactive career strategies to navigate life challenges (Lo Presti et al., 2019), potentially leading to a lack of the need to develop these resources further.

Broader external factors also need consideration. SCCT indicates that employability resources depend on dynamic interactions between individual features (e.g., gender), personal resources (e.g., outcome expectations), and external factors (e.g., job market) (Lent and Brown, 2013). Control group participants, by virtue of not receiving the intervention, may have lacked the supportive framework and resources to navigate both their internal resources and external influences effectively. Without targeted guidance and resource-building activities, they may have been more susceptible to the negative impacts of external factors such as economic downturns, limited job opportunities, and societal pressures (Lent et al., 2022). Additionally, the absence of proactive career strategies emphasised in the intervention may have left control group participants less equipped to adapt to changing market demands or capitalise on available opportunities (Lo Presti et al., 2019). This suggests a need for comprehensive research that addresses both individual characteristics and contextual factors influencing employability of unemployed adults (Blustein et al., 2020; Thompson et al., 2017).

When analysing the effects of being part of the intervention group, it appears that participants in both the face-to-face and online modalities did not experience the same decline as those in the control group. Instead, they showed a more positive trajectory in employability resources over time. Therefore, while the general trend showed a decline in employability resources among all participants, being part of either intervention group served as a protective factor against this decline, resulting in positive changes in employability resources over time for intervention participants. These results align with expectations regarding the effectiveness of career psychological interventions for unemployed clients (e.g., Maree, 2022). By addressing the pervasive challenges of unemployment, these interventions offer a proactive solution to empower individuals with the resources and support needed to enhance their employability (Lo Presti and Pluviano, 2015; Panari et al., 2020). Empowerment can increase individuals’ chances of developing proactive career strategies (Lo Presti et al., 2022), and have broader societal impacts, such as significant economic benefits from investing in high-quality career development programs (Carvalho et al., 2024). Empirical guidelines on intervention design and evaluation (Lent and Brown, 2020), along with previous research (Carvalho et al., 2024), should serve as foundational resources for designing career interventions and ensuring their effectiveness.

Moreover, it is noteworthy that both face-to-face and online intervention modalities produced similar positive effects on employability resources, supporting the idea that online interventions can be as effective as traditional face-to-face interventions (e.g., Amundson et al., 2018). The success of this intervention highlights the effectiveness of adaptations made to the original face-to-face intervention. To ensure quality parameters of online interventions, adaptations such as reducing session quantity and duration were implemented (Pordelan et al., 2018; Sampson et al., 2019). Selecting appropriate applications or platforms that support secure communication and ensuring data privacy and protection were crucial steps in facilitating the transition to online interventions (American Psychological Association, 2013; Ordem dos Psicólogos Portugueses, 2020). Establishing guidelines to maintain a private and distraction-free environment during sessions should also be considered to optimise the therapeutic process and ensure confidentiality (Ordem dos Psicólogos Portugueses, 2020). These measures collectively demonstrate that with careful planning and implementation, both face-to-face and online interventions can be equally effective. This highlights the advantages of incorporating online modalities to enhance accessibility and participation (Pordelan and Hosseinian, 2020), while also acknowledging the continued benefits of traditional face-to-face interventions, particularly in fostering personal connections (Carvalho et al., 2024). Moreover, the effectiveness of the intervention can also be ascribed to the training received by the psychologists who implemented it, highlighting the crucial role of training professionals for this type of initiative (Blustein et al., 2019).

Although the LGC analysis did not distinguish specific growth tendencies between time points, the significant positive growth tendency suggests that the intervention had a lasting impact on participants’ employability resources, indicating its promising long-term effectiveness. This underscores the potential of psychological interventions to foster sustained improvements in employability outcomes, extending beyond the immediate post-intervention period (Perdrix et al., 2012). However, it’s not possible to guarantee that the growth in employability resources among the study participants led to re-entry into the job market or educational outcomes, as seen in other studies (Littman-Ovadia et al., 2014). Nonetheless, it can be speculated that given the employability positive curve, intervention participants are more likely to engage in job search behavior (Lo Presti and Pluviano, 2015). Further insights into intervention sustainability will be gained through the analysis of each dimension’s strength at the follow-up moment, identifying participants’ additional needs, and providing opportunities to enhance future interventions (Sadler, 1984).

At the beginning of the study, Human Capital and Professional Development (HCPD) was the employability dimension that better explained global employability resources, followed by Social Capital and Networking (SCN), Environmental Monitoring (EM), and finally Career Identity and Self-Management (CISM). This suggests that participants already initially placed a strong emphasis on their existing perceptions of knowledge, skills, and competencies, and in recognising the importance of social relationships and networks as key determinants of their employability (Lo Presti et al., 2019). Even before any stimulus, individuals already seemed to be aware of the importance of staying informed about changes in the labour market, industry trends, and potential employment opportunities as something very important to enhance their employability (Lent et al., 2022). Furthermore, awareness of one’s own career resources was seen as less important in explaining participants’ employability, which is consistent with the expected lack of career self-awareness of unemployed individuals who may not have the confidence and opportunity to reflect on their career values, interests and goals (Taveira et al., 2017).

After the intervention, the Social Capital and Networking (SCN) dimension emerged as the most influential in explaining global employability resources, closely followed by the Human Capital and Professional Development (HCPD) dimension. This shift suggests that the intervention likely promoted social interactions and the recognition of career networks among participants, allowing them to leverage social capital for career advancement (Carvalho et al., 2024). The emphasis on building social relationships and expanding career networks within the intervention likely promoted participants’ employability during the post-test period, highlighting the crucial role of social support for individuals facing unemployment (Bhat, 2010; Lent et al., 2022). Furthermore, the recognition of career goals and the ability to develop an individual career action plan emerged as significant factors in explaining participants’ employability following the intervention (e.g., Drosos et al., 2021; Rezaei et al., 2017). Participants’ drive to achieve their career aspirations, supported by their ability to envision their next steps and implement their career plans, likely played a fundamental role in this outcome. The structured approach to career planning provided by the intervention appeared to empower participants to take proactive steps towards their career goals (Carvalho et al., 2024). Furthermore, the ability to interpret one’s context and navigate environmental factors (Lo Presti et al., 2019) emerged as another important contributor to participants’ employability post-intervention. This is consistent with the intervention’s focus on enhancing participants’ environmental monitoring skills, which appeared to resonate with participants and positively impact their employability outcomes. Participants demonstrated an increased ability to explore and understand their professional environment, enabling them to adapt their career strategies accordingly (Littman-Ovadia et al., 2014). However, while participants demonstrated an increased awareness of their career identity compared to previous points in time, this dimension remained less salient in explaining overall employability. This should simultaneously highlight the power of career interventions to stimulate self-awareness (Drosos et al., 2021), but also the demands of developing one’s career and employability through self-reflection (e.g., Maree, 2022).

Two months after the intervention, the post-intervention pattern remained consistent, with slightly higher loadings observed for every dimension except CISM. In the follow-up assessment, SCN and HCPD were the dimensions that most significantly explained global employability resources, followed by EM, and lastly CISM. This suggests that at a long-term level, the effects of the intervention persisted, with certain dimensions exhibiting sustained influence on participants’ employability. The role of social networks continued to be highly significant for participants even two months post-intervention. This unexpected finding underscores the enduring impact and importance individuals in vulnerable situations place on their social networks (Lent et al., 2022; Taveira et al., 2017). Similarly, participants’ commitment to career planning activities persisted beyond the immediate post-intervention period. The establishment of long-term career plans during the intervention likely appeared to foster a sense of commitment among participants, leading to continued engagement in career development activities (Carvalho et al., 2024). This aligns with the expectation that participants would remain focused on their career goals after the intervention (Drosos et al., 2021; Littman-Ovadia et al., 2014). Furthermore, the sustained ability to interpret one’s environment and adapt career strategies accordingly highlights the effectiveness of the intervention in fostering long-term skills development (Taveira et al., 2017). Participants’ proactive engagement in monitoring labour market dynamics likely contributed to their sustained employability outcomes over time. Following the same pattern as the previous time points, individual resources that promote career self-awareness and enable one to make sense of past experiences and anticipate the future (Lo Presti et al., 2019) remained the less powerful dimension explaining participants’ employability. This suggests that while career self-awareness is important for career development (Cheng et al., 2020), in the longer term, other practical tasks such as career planning and networking might prevail, especially in a situation of unemployment where individuals are faced with numerous challenges and competing priorities (Drosos et al., 2021). It is therefore understandable that participants may prioritise more immediate and tangible tasks that directly contribute to their career success (Lo Presti et al., 2019), such as developing a career plan and expanding their social networks.

## 5 Limitations and future directions

Despite the valuable insights gained from this study, several limitations warrant consideration and suggest avenues for future research. Firstly, it is important to consider some factors related to the study sample that may influence the results. The major innovation of using a longitudinal sample also posed some challenges. Due to sample limitations, especially at the follow-up assessment, the study’s measurement instruments may not have captured employability resources as accurately as expected. Future research designs should use strategies to retain study participants after the intervention (e.g., Poongothai et al., 2023). In addition, assuming the sample size can be maintained, longer-term follow-up assessments beyond the 2-month period could provide a more comprehensive understanding of the ongoing effectiveness of the intervention. Furthermore, results may be influenced by sample demographic characteristics. In line with SCCT principles (Lent and Brown, 2013), future studies should explore how factors such as age, gender, level of education, and previous and current work and training experience interact with intervention outcomes. In addition, process and qualitative information should be considered, as this is considered critical to understanding how participants engage with the intervention components, identifying potential areas for improvement and ensuring fidelity to the intervention protocol (Lent and Brown, 2020).

Secondly, although this study presents a robust solution for evaluating the intervention using a latent growth model, which has been recommended for the career intervention research line (Spurk et al., 2020), there were some limitations to highlight. Due to the sample size, it wasn’t possible to include more variables in the model (e.g., socio-demographic, other career variables such as life satisfaction, perceived social support, career self-efficacy, etc.), which could have helped to better understand the employability trajectory. Additionally, the sole reliance on self-report measures limits the robustness of the findings. Future research should incorporate objective data, such as employment status post-intervention, how the employment status changed, and satisfaction with new roles, to provide a more comprehensive evaluation of the intervention’s impact. Finally, future research could also explore sensitivity analyses to further validate the robustness of the findings.

## 6 Conclusion

The study aimed to evaluate the effectiveness of a career intervention for unemployed adults within the framework of Social Cognitive Career Theory (SCCT). By analysing changes in employability resources over three time points and comparing the effects of intervention modalities on employability trajectories, several key findings emerged. There was a general decline in employability resources among participants, highlighting the pervasive challenges faced by the unemployed. Despite this decline, both intervention groups showed positive trajectories. By focusing on developing skills and resources across multiple dimensions, interventions can be effective in improving overall employability and supporting individuals to manage career transitions and challenges. The success of career interventions also highlights the need to invest in high-quality programmes and adhere to empirical guidelines. In addition, both face-to-face and online interventions showed similar positive effects, highlighting the adaptability of online modalities and reinforcing the effectiveness of traditional face-to-face modalities. The positive growth trend observed suggests a lasting impact of the intervention on employability resources, while further research is needed to understand long-term sustainability and outcomes. The shift in influential dimensions at the three time points showed that prior to any intervention stimuli, participants appeared to prioritise tangible skills and social connections in their perceptions of employability resources. The intervention could have focused more explicitly on improving social connections and professional skills. In the longer term, it highlighted the need for further intervention in participants’ self-awareness to ensure comprehensive support for participants in all aspects of their employability journey. Overall, the study provides valuable insights into the effectiveness of career interventions for unemployed adults, offering an innovative research design and statistical approach, proactive solutions with potential societal impact, and highlighting the need for further research to refine intervention strategies and maximise effectiveness in supporting individuals’ careers.

## Data Availability

The raw data supporting the conclusions of this article will be made available by the authors, without undue reservation.
